# The impact of environmental regulations on the location choice of newly built polluting firms: based on the perspective of new economic geography

**DOI:** 10.1007/s11356-022-19956-8

**Published:** 2022-04-08

**Authors:** Na Peng, Xiangjian Zhang

**Affiliations:** School of Urban and Regional Science, Shanghai University of Finance and Economics, Shanghai, 200433 China

**Keywords:** Environmental regulations, Newly built polluting firms, Location choice, New economic geography

## Abstract

Based on the unique micro-data of newly built polluting firms for the period of 2009–2018, this paper adopts the conditional logit model to empirically evaluate the impact of environmental regulations on the location choice of polluting firms. Moreover, we extend the theoretical model by considering that the environment regulations not only influence the pollution cost but also the level of technological innovation and labor cost. The empirical results show that polluting firms tend to flow into areas with stringent environmental regulations, which supports the Porter hypothesis, but the effect of environmental regulations have a divergent impact on heavily polluting firms. Heterogeneous analysis indicates that environmental regulations have shown a positive impact on the location choice of private and foreign-funded firms but no significant impact on that of state-owned firms; the impact of environmental regulation is consistent with pollution haven hypothesis for firms in the central region but is in line with Porter hypothesis for firms in other regions. Meanwhile, the probability of air polluting firms entering areas with stricter environmental regulations is higher than that of water-polluting ones. Finally, this paper further empirically tests the conduction mechanism, that is, environmental regulations can affect the location choice of polluting firms by affecting the regional technological innovation capabilities and labor cost.

## Introduction

With the rapid economic growth, China’s environmental problems have become increasingly prominent and have attracted great attention from the government and the public (Wang et al. 2015). Starting from the 9th Five-Year Plan for National Economic and Social Development, the central government has issued quantitative environmental policies, taking a significant reduction in the total discharge of major pollutants as a binding indicator for social and economic development. Since the start of the 11th Five-Year Plan, the total control targets for sulfur dioxide and chemical oxygen demand have been achieved ahead of schedule, but the problems of air and water pollution remain severe. The main reason is that the central government has delegated greater autonomy to local governments in terms of environmental policies and economic development since the 1980s. Specifically, the central government sets an overall pollutant emission reduction target and then distributes the target to each province. Each province formulates its own pollution control target based on local economic level, industrial structure, emission reduction potential, environmental bearing capacity, and development plan. Because of the spatial variation of economic and social development, there are huge differences in environmental standards among regions, and it is getting lower from the eastern region to the western region (Yin et al. 2015; Dou and Han 2019; Wang et al. 2019). The lax environmental policies in the central and western regions will attract the inflow of polluting firms. Therefore, although the overall pollutant emissions are reduced, the pollutants are gradually transferred to areas with poor environmental governance capabilities, which may lead to the deterioration of the overall ecological environment (Cai et al. 2016).

Since pollution reduction tasks are delegated to local governments, the implementation standards of environmental policies among regions are different (Zhou et al. 2017), which will not only lead to the transfer of polluting industries, but also affect the location choice of new-built polluting firms. Will relatively lax environmental policies attract firms to enter and stricter one prevent so? Many scholars have conducted a lot of research on this issue, and mainly formed two opposing hypotheses, namely the “Porter hypothesis” and the “pollution haven hypothesis.” The former believes that appropriate environmental regulations can encourage firms to innovate and increase their market competitiveness; the improvement of firm productivity can make up for the cost of compliance (Poter and Linde 1995). While the latter holds that strict environmental regulations will increase the production costs of firms and reduce their market competitiveness, so firms will choose to move to areas with relatively loose environmental regulations for the seeking of profits (Ambec et al. 2013).

In recent years, many scholars have empirically tested the impact of environmental regulations on the location of firms. Some scholars believe that strict environmental regulations are attractive to firms (Ben Kheder and Zugravu 2012; He et al. 2014; Shao et al. 2019). A study found that Japanese heavily polluting firms tend to invest in countries and regions with more stringent environmental regulations (Kirkpatrick and Shimamoto 2008). Some scholars have found that the impact of environmental regulations on the location choice of firms is heterogeneous, and firms in the eastern region are more willing to invest in provinces with stricter environmental regulations (Wang et al. 2019). Dou and Han (2019) believe that firms with poor liquidity tend to innovate locally instead of migrating to other regions while faced with stricter environmental supervision. On the contrary, some scholars support the pollution haven hypothesis and believe that loose environmental regulations will attract the inflow of firms (Sarkodie and Strezov 2019; Yuan et al. 2019). Mulatu et al. (2010) took data from 13 EU countries to conduct research and found that loose environmental regulations will attract polluting firms to enter. Using the firm migration data of Guangdong province, Shen et al. (2017) have found that more stringent environmental regulations have prompted polluting firms in the Pearl River Delta to move to the surrounding areas and that the introduction and strict enforcement of environmental regulations can prevent areas from becoming “pollution haven.” Wu et al. (2017) used the data of newly built polluting firms in China from 2006 to 2010 and found that the location choice of these firms has changed from the eastern region with strict environmental regulations to the central and western regions with loose environmental regulations. However, some scholars believe that environmental regulations have not played a significant role in the location choice of firms (Levinson 1996; Manderson and Kneller 2012; Mulatu and Wossink 2014). Recent studies focus on heterogeneous analysis, and the results indicate that the effectiveness of environmental regulations depends on the types of environmental regulations, industry and firm characteristics, and resource endowments (Zheng and Shi 2017; Zhou et al. 2017; Cai et al. 2018) and that the “Porter effect” and “pollution haven effect” of environmental regulations can coexist (Zhou et al. 2017; Wu et al. 2019).

The main reason for the huge difference in empirical results is that most studies use aggregated data on economic activities at the industry or city level, such as net investment, the number of existing or newly built firms, and employment growth. Aggregated data cannot tell whether the impact of environmental regulations is due to the building of new firms or the expansion or contraction and closure of existing firms (Levinson 1996; Mulatu et al. 2010; Mulatu and Wossink 2014; Shen et al. 2017; Cai et al. 2016; Wu et al. 2019).

Based on the *List of National Key Monitoring Firms* and *List of Key Pollutant Discharge Units*, this paper has compiled data on newly built polluting firms from 2009 to 2018. Using unique micro-firm data and the conditional logit model to study the impact of environmental regulations on the location choice of polluting firms, we can effectively avoid the bias caused by the use of aggregated data. We find that polluting firms tend to flow into areas with stricter environmental regulations except for heavily polluting firms. Meanwhile, this paper conducts a heterogeneous analysis based on different regions, different pollutants, and different ownerships and finds that environmental regulations have different impacts on the location choice of different polluting firms.

Compared with the existing literature, the marginal contribution of this paper mainly lies in the following three aspects: (1) most of the existing studies on the location choice of firms are based on samples of the Chinese industrial firm database. However, this paper has compiled unique data on newly built polluting firms from 2009 to 2018. Compared to the dataset of Chinese industrial firms, the data are not only newer, but also distinct newly built air polluting firms and water-polluting ones. At the same time, for avoiding the measure error of technological progress, the patent data was obtained from the patent search and service platform of the China National Intellectual Property Administration. (2) Based on the new economic geography theory, this paper adds environmental regulation factors to the previous theoretical models and explores the influence of environmental regulation on the location choice of firms through the model instead of merely qualitative analysis. (3) This paper further explores the mechanism of environmental regulations affecting the location choice of polluting firms through model derivation; we extend the theoretical model by considering that the environment regulations not only influence the pollution cost but also the level of technological innovation and labor cost.

The remainder of the paper is organized as follows. Section 2 is theoretical analysis. Section 3 introduces the methodology. Section 4 reports the empirical results. Section 5 specifies the mechanism test, and Section 6 is reserved for the conclusion.

## Theoretical analysis

The new economic geography theory believes that firms tend to choose locations in areas with greater market potential, mainly because such area has a favorable business environment, strong technology spillover effects, and perfect infrastructure conditions, etc. However, in areas with greater market potential, the environmental problems caused by economic growth are more severe, and the corresponding environmental regulatory measures are also stricter, which will increase the compliance cost of firms. According to the Porter hypothesis, appropriate environmental regulations can stimulate technological innovation and cleaner production and further increase their productivity and competitiveness, thereby offsetting the costs of complying with them.

This paper draws on the theoretical model developed by Ben Kheder and Zugravu (2012). In this model, pollution is considered as a third production factor, together with labor and capital. We extend the model by considering that the environment regulation not only influences the pollution cost but also the level of technological innovation and labor cost. The general assumption of this economic geography model is there are two production sectors in a small open economy, namely, agriculture and industry. The former produces homogeneous commodities under Walrasian equilibrium conditions, and the latter produces heterogeneous commodities under the condition of increasing returns to scale in the environment of monopoly competition. The elasticity of substitution between different products is $\sigma >1$, and the transportation cost of the products between the two regions is $\tau$.

We write the profitability *U* of a firm *h* located in area *i* and trading with other area *j*:

1 $${U}_{i}\left(h\right)=ln{MP}_{i}-\left(\sigma -1\right)ln{c}_{i}(h)$$

where ${MP}_{i}={\sum }_{j}{\tau }_{ij}^{1-\sigma }(\mu {E}_{j}/{G}_{j})$ is the market potential; ${G}_{j}={\sum }_{i}{n}_{i}{[{c}_{i}\left(h\right){\tau }_{ij}]}^{1-\sigma }$ indicates the competitiveness of firms from other areas; ${E}_{j}$ measures the total expenditure on commodities in other area $j$; $\mu$ is the expenditure share of different commodities; ${c}_{i}\left(h\right)$ is the marginal cost of producing commodity $h$ in area $i$.

In the model of Ben Kheder and Zugravu (2012), the cost function adopts the most common Cobb–Douglas function and meanwhile includes pollution as a production cost: $c=(1/A){w}^{\alpha }{r}^{\beta }{t}^{\theta }$, where $\theta =1-\alpha -\beta ,\;and\;w,\;r\;and\;t$ represent labor cost, capital cost, and pollution cost respectively, and $\alpha$, $\beta$, and $\theta$ represent the share of labor, capital, and pollution factors in the production process of a firm, whereas $A$ is the level of technological innovation. Meanwhile, we assume that the technological level of firms and labor cost will be affected by environmental regulations $e$, and the marginal cost function of firm $h$ located in area $i$ can be expressed as:

2 $${c}_{i}\left(h\right)=\frac{1}{{A}_{i}({e}_{i})}{w}_{i}{\left({e}_{i}\right)}^{\alpha }{{r}_{i}}^{\beta }{t}_{i}{\left({e}_{i}\right)}^{\theta }{\varphi }_{i}$$

where ${\varphi }_{i}$ are other factors that affect the marginal cost of the firm, including land resources, market competitiveness, and transportation costs. Since the level of regional environmental regulations can promote the technological innovation ability of firms and increase the pollution control cost of firms, we can get $\partial {A}_{i}({e}_{i})/\partial {e}_{i}>0$ and $\partial {t}_{i}({e}_{i})/\partial {e}_{i}>0$. Some studies have found that environmental regulation will reduce the actual wage level of firms. On the one hand, with the increasing intensity of environmental regulation, polluting industries will begin to limit production or even withdraw from the market, leading to a decrease of the average wage. On the other hand, the production cost and pollution control cost of firms facing environmental regulation will increase, and firm’s profits will decrease under the condition that the market demand remains unchanged; thus, firms will reduce the real wage. Therefore, we can get $\partial {w}_{i}({e}_{i})/\partial {e}_{i}<0$.

By introducing Eq. (2) into Eq. (1), we can write Eq. (1) in the following way:

3 $${U}_{i}\left(h\right)=\mathrm{ln}{MP}_{i}+\left(\sigma -1\right)\mathrm{ln}{A}_{i}\left({e}_{i}\right)-\alpha \left(\sigma -1\right)\mathrm{ln}{w}_{i}\left({e}_{i}\right)-\beta \left(\sigma -1\right)\mathrm{ln}{r}_{i}-\theta \left(\sigma -1\right)\mathrm{ln}{t}_{i}\left({e}_{i}\right)-\left(\sigma -1\right)\mathrm{ln}{\varphi }_{i}$$

We assume that environmental regulation will not affect the market potential and calculate the partial derivatives of ${e}_{i}$ on both sides of Eq. (3). We can get the partial derivatives of the firm’s profitability as follows:

4 $$\frac{\partial {U}_{i}\left(h\right)}{\partial {e}_{i}}=\left(\sigma -1\right)\left(\frac{\dot{{ A}_{i}}}{{A}_{i}}-\alpha \frac{\dot{{ w}_{i}}}{{w}_{i}}-\theta \frac{\dot{{t}_{i}}}{{t}_{i}}\right)$$

Equation (4) predicts that the profitability of a firm *h* settled in a province* i* is an increasing function of technological innovation capability and a decreasing function of pollution control costs and labor cost. We can know that environmental regulations can affect the location choice of firms by influencing the technological level and labor cost.

Based on the above analysis, we know that the improvement of environmental regulation standards will increase the production costs of firms in the short term, but it can force firms to promote the technological innovation level and cut labor cost in the long run, which will help firms to internalize the additional costs for compliance (Rubashkina et al. 2015; Wu et al. 2019). Therefore, polluting firms are more likely to enter areas with stricter environmental regulations. However, for heavily polluting firms, it is difficult to evade environmental supervision, and the improvement of environmental regulation standards will significantly increase the pollution control costs and squeeze investment in technology research and development. Therefore, heavily polluting firms are more inclined to flow into areas with relatively loose environmental regulations. This specification represents the theoretical background for our following empirical work.

## Methodology

### Conditional logit model

This paper uses the conditional logit model proposed by McFadden (1974) to evaluate links between environmental regulations and firm location choice. This model is a discrete choice model based on the goal of profit maximization and is usually used to study the problem of firm location choice (Levinson 1996; Ben Kheder and Zugravu 2012; Wu et al. 2017; Wang et al. 2019). In this model, firms will compare the profits in different regions and then choose to invest in the regions where the profits are maximized.

Assuming that the polluting firm $h$ pursues the maximization of profit, the profit function of the firm $h \mathrm{located in} \mathrm{ province }j$ at year $t$ is given by:

5 $${\pi }_{hjt}=\beta {ER}_{jt}+\delta {X}_{jt}+{\varepsilon }_{hjt}$$

where ${ER}_{jt}$ is the environmental regulation level of province $j$ at year $t$;${X}_{jt}$ is the observable characteristic variable of province $j$ at year $t$;${\varepsilon }_{hjt}$ is a random error term, which is assumed to be independently and identically distributed. The probability that the polluting firm $h$ choose province $j$ out of $k \mathrm{possible}$ provinces at year $t$ is:

6 $$Prob\left({Y}_{hjt}=1\right)=\frac{\mathrm{exp}(\beta {ER}_{jt}+\delta {X}_{jt}+{\varepsilon }_{hjt})}{{\sum }_{1}^{k}\mathrm{exp}(\beta {ER}_{jt}+\delta {X}_{jt}+{\varepsilon }_{hjt})}$$

where $k$ equal to 30 includes all the provincial administrative regions except Tibet, where there are fewer newly built polluting firms.

In order to explore the difference in the impact of environmental regulations on the location choice of firms with different pollution intensities, this paper adds an interaction term between environmental regulations and firm pollution intensities in the regression equation. The economic model is set as follows:

7 $$Prob\left({Y}_{hjt}=1\right)=\beta {ER}_{jt}+\theta {ER}_{jt}*{PI}_{ht}+\delta {X}_{jt}+{\omega }_{j}+{\varepsilon }_{hjt}$$

where ${Y}_{hjt}$ equals to 1 if the polluting firm $h$ is located in the province $j$ at year $t$ and 0 otherwise; the coefficient $\beta$ measures the average effect of environmental regulations on the location choice of the newly built polluting firms; ${PI}_{ht}$ indicates the pollution intensity of firm $h$; this paper adopts the classification criteria for heavy pollution industries in the *First National Pollution Source Census Plan* issued by the General Office of the State Council in 2007 and selects 11 heavy-polluting industries.^1^ If the polluting firm belongs to the heavily polluting industry, ${PI}_{ht}$ equals to 1 and 0 otherwise. The coefficient θ measures the effect of environmental regulations on the location choice of newly built firms with different pollution intensities. ${\omega }_{j}$ is the regional fixed effect, which captures all time-invariant differences across the region such as geographical location, climate, and resource conditions. ${\varepsilon }_{hjt}$ is the error term.

### Sample selection

To conduct the research, we construct a sample with firm-level location data from the 2009–2017 *List of National Key Monitoring Enterprises* and the 2018–2020 *List of Key Pollutant Discharge Units*. According to the *Instructions of List of National Key Monitoring Enterprises* issued by the State Environmental Protection Administration in 2007, these lists include wastewater firms, waste gas firms, and sewage treatment plants and report the name and location of each firm, of which the emissions accounted for more than 65% of all firm emissions. With the continuous deepening and refinement of environmental supervision measures, the Ministry of Environmental Protection issued the *Regulations on the List of Key Pollutant Discharge Units (Trial)* in 2017 as the replacement and continuation of the *List of National Key Monitoring Enterprises*. The *List of Key Pollutant Discharge Units* covers wider, including five types of key pollutant discharge units: key pollutants discharge unit of water environment, atmospheric environment, soil environmental, acoustic environment, and others.

Since the main targets of the wastewater treatment plants are municipal sewage treatment, and its location decision is generally the outcome of government interference rather than free choice, this paper excludes wastewater treatment plants in the analysis. In both lists, the province where the firm is located is publicized, so it’s easy to obtain the location information of the polluting firm, and the year of establishment of the firms can also be traced to form required sample data of newly built polluting firms. The reason for choosing a newly built polluting firm is that existing firms have fixed costs. Only when the costs of environmental compliance exceed the costs of relocation of the firm, it will choose to relocate. Therefore, the fixed costs of existing firms make them insensitive to the implementation of environmental regulations, and the use of newly built firms without sunk costs can effectively avoid this problem (Levinson 1996).

**Fig. 1:**
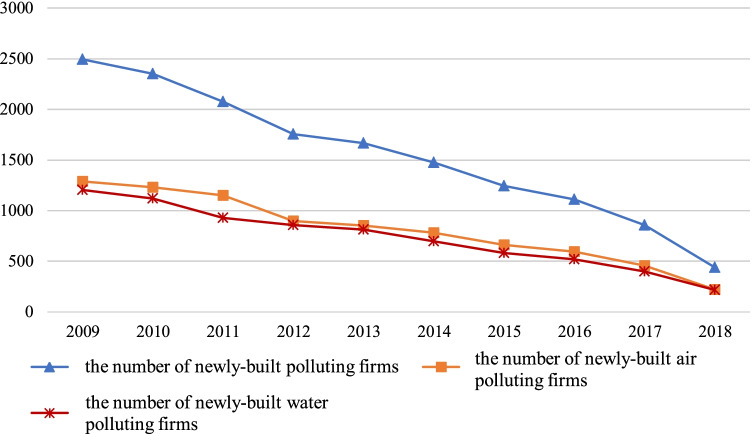
The dynamic trends of newly built polluting firms, 2009–2018. Data source: *List of National Key Monitoring Firms* 2009–2017 and *List of Key Pollutant Discharge Units* 2018–2020

Some firms may have path dependence when making location selection instead of aiming at maximizing profits. For example, a subsidiary may be located at the location of its parent company. The firms as subsidiaries are excluded in the data processing to reduce the estimation error. Through sorting out and deleting firms with missing information and recurring information, we finally obtain 15472 valid samples. Table 1 and Fig. 1 show the regional distribution and dynamic trends of newly built polluting firms, respectively.

**Table 1:** The regional distribution of newly built polluting firms

Region	2009	2010	2011	2012	2013	2014	2015	2016	2017	2018
Eastern	1452	1453	1300	1109	1085	933	804	735	539	249
Central	610	498	435	409	370	328	280	243	227	130
Western	432	399	341	238	213	217	161	133	90	59
Total	2494	2350	2076	1756	1668	1478	1245	1111	856	438

List of National Key Monitoring Firms 2009–2017 and List of Key Pollutant Discharge Units 2018–2020.

It can be seen from Table 1 that the spatial distribution of the number of newly built polluting firms is extremely unbalanced: the number is gradually decreasing from the eastern region to the central and western regions, with significant gradient differences. Among them, the number of newly built polluting firms in the eastern region accounted for more than a half.

As shown in Fig. 1, the number of newly built polluting firms has shown a dynamic characteristic of gradual decline. Especially, the number has dropped sharply after 2016. The main reason may be that the Chinese government began to implement the revised *Environmental Protection Law* since 2015. This law reflects unprecedented environmental protection and governance efforts from multiple angles, known as the “strictest” environmental protection law in history. The implementation of environmental policies can reduce the number of polluting firms, but the effectiveness of policies is time-lagged.

### Variable measure

Dependent variable: firm location choice $(Choice)$. If a firm makes more profit by choosing province *j* to locate at year *t*, then ${Choice}_{hjt}=1$; otherwise, it is 0.

8 $${Choice}_{hjt}=\left\{\begin{array}{cc}1,& \mathrm{if} {\pi }_{hjt}>{\pi }_{hkt},\forall j\ne k\\ 0,& \mathrm{other}\end{array}\right.$$

Independent variable: environmental regulations (*ER*). It has remained controversial as for how to accurately measure environmental regulations. Existing studies have mostly used indicators such as pollution discharge fees, pollution control investment, and pollutant removal rate to describe the degree of implementation of environmental regulations (Wang et al. 2019), but these indicators could hardly reflect the degree of implementation of environmental regulations accurately. Therefore, this paper uses the proportion of the number of environmentally illegal firms investigated by the government to the number of industrial firms to measure the degree of implementation of environmental regulations. The data of environmentally illegal firms come from the corporate environmental information database published by the Institute of Public and Environmental Affairs (IPE). The IPE sorts information of environmentally illegal firms released by the government to establish a database consisting of the name of such enterprises, type of and reason for the violation, and means and time of punishment. The type of the violation mainly includes air pollution, water pollution, solid waste pollution, and noise pollution. The data of the IPE basically come from the information investigated by the local environmental protection bureau, but the channels for obtaining relevant information are not limited to local government websites, but also platforms such as the media, the website of the Provincial Department of Environment, and the website of the Ministry of Environmental Protection. Therefore, the environmental regulation indicators adopted in this article are not completely affected by the degree of local government environmental information disclosure, and thus the resulting bias may be small. The data of industrial firms comes from the *China Industrial Statistical Yearbook*.

Control variable: in order to reduce the estimation bias caused by the omitted variables, some control variables at the provincial level are added to the regression model. The selection of control variables is mainly based on the factors affecting location choice in neoclassical theory and new economic geography theory. The data mainly come from the *China Statistical Yearbook*, *Urban and Rural Construction Statistical Yearbook*, etc.

Market size: the potential market size is captured by per capita gross national product (*pgdp*) and population (*pop*). The data come from the *China Statistical Yearbook.*

Human capital: the scale of labor (*labor*) is defined as the proportion of the population aged 15–64; the quality of labor (*illiterate*) is reflected by the illiteracy rate of the population aged 15 and above; and the average wage of employees is used to measure the labor cost (*wage*). The data come from the *China Statistical Yearbook.*

Infrastructure: the traffic accessibility and transportation costs are expressed by the length of railway transportation lines (*railway*). The data come from the *China Statistical Yearbook.*

Other influencing variables: the impact of land policies is measured by area of construction land (area) as an alternative indicator of land supply; the proxies for the level of technological innovation we use is the research and development expenditure (*rd*); the market competition from foreign investors is defined by foreign direct investment (*fdi*). The data of the level of technological innovation and foreign direct investment come from the *China Statistical Yearbook.* The data of area of construction land come from the *Urban and Rural Construction Statistical Yearbook* (Tables 2 and 3).

**Table 2:** Definition and measurement of variables

Variables		Symbol	Definition	Measurement
Dependent variable		*Choice*	Firm location choice	If the polluting firm $h$ chooses province $j$ at year $t$, then *Choice* = 1; otherwise, it is 0
Independent variable		*ER*	Environmental regulation	Proportion of environmentally illegal firms to the total industrial firms
		*PI*	Pollution intensity	If a firm is a heavily polluting firm, then PI = 1; otherwise, it is 0
Control variables	Market size	*pgdp*	Economic level	Per capita GDP
		*pop*	Population	Total population at the end of year
	Human capital	*labor*	Scale of labors	Proportion of people aged 15–64
		*illiterate*	Quality of labors	Illiteracy rate of the population aged 15 and above
		*wage*	Labor cost	Average wage of employees
	Infrastructure	*railway*	Traffic accessibility	Length of railway transportation lines
	Other influencing variables	*area*	Land policy	Construction land area
		*rd*	Technological innovation	Research and development expenditure
		*fdi*	Market competition from foreign investors	Foreign direct investment

Own elaboration.

**Table 3:** Descriptive statistics of variables

Variables	Number	Mean	Sd	Min	Max
*Choice*	464160	0.0333	0.1795	0	1
*ER*	464160	0.1197	0.1695	0.0020	0.8623
*PI*	464160	0.2256	0.4180	0	1
ln*pgdp*	464160	10.5679	0.4952	9.3030	11.8509
ln*pop*	464160	8.1865	0.7393	6.3226	9.3366
*labor*	464160	0.7410	0.0362	0.6621	0.8385
*illiterate*	464160	0.0533	0.0301	0.0123	0.1663
ln*wage*	464160	10.7328	0.3430	10.1144	11.9173
ln*railway*	464160	7.9330	0.6876	5.7611	9.4545
ln*area*	464160	7.0891	0.7714	4.7185	8.6265
ln*rd*	464160	13.8974	1.3642	9.5782	16.8713
ln*fdi*	464160	5.3585	1.6060	0.3438	7.7219

Own elaboration

## Empirical results

### Main results

Table 4 reports the baseline estimation results concerning the impact of environmental regulations on the location choice of newly built polluting firms. Since the location choice of a firm is a multi-valued variable, it is suitable to use the conditional logit model. The conditional logit model effectively alleviates the problem of missing variables at the individual level, but the factors that affect the location of polluting firms are complex, and there are likely other unobservable factors that lead to estimation errors. In order to solve the endogenous problems, this paper adopts an instrumental variable strategy following Hering and Poncet (2014), who use the ventilation coefficient (*vc*) as an instrumental variable of the environmental regulations. The ventilation coefficient is equal to the wind speed by the height of the boundary layer. The ERA-Interim database of the European Meteorological Center (ECMWF) provides raster data of wind speed at a 10-m height and boundary layer height (75 × 75). This paper first calculates the ventilation coefficient of each raster corresponding to the year and then matches the raster data with our Chinese provinces according to their latitudes and longitudes to obtain the ventilation coefficient of each province in the sample period 2009–2018 (Shi and Xu 2018; Cai et al. 2016).

**Table 4:** Main estimation results

Variables	The first stage (*ER*)	The second stage (*Choice*)
*vc*	− 0.0142***	
	(0.0203)	
*ER*		3.4783***
		(0.8291)
ER*PI		− 5.7463***
		(1.1983)
ln*pgdp*		1.1784*** (0.0573)
ln*pop*		0.8920*** (0.0745)
*labor*		− 8.1901*** (0.5214)
*illiterate*		− 0.8371*** (0.4681)
ln*wage*		− 1.6793*** (0.1173)
ln*railway*		0.6391*** (0.0194)
ln*area*		0.0842 (0.0573)
ln*rd*		0.2298*** (0.0372)
ln*fdi*		0.3571*** (0.0082)
Regional fixed effect	Yes	Yes
_*cons*	0.0833	
	(0.0723)	
*N*	300	464,130
Log likelihood		− 49,824
Prob > chi2	0.0000	0.0000

***, **, and * indicate significant under 1%, 5%, and 10% levels, respectively; figures in the parentheses are standard error.

Own elaboration.

Based on this instrumental variable, this paper uses two-stage least squares estimation (2SLS) to conduct the regression. In the first stage, we take environmental regulation as a dependent variable to regress the ventilation coefficient to obtain the fitted value $\widehat{ER}$ of the environmental regulations, namely:

9 $${ER}_{jt}={\alpha }_{1}{vc}_{jt}+{\omega }_{j}+{\gamma }_{t}+{\varepsilon }_{jt}$$

In the second stage, the fitted value $\widehat{ER}$ obtained in the first stage is used as the main independent variable, namely:

10 $${Choice}_{hjt}={\varphi }_{1}\widehat{{ER}_{jt}}+{\varphi }_{2}\widehat{{ER}_{jt}}*{PI}_{ht}+{\varphi }_{3}{X}_{jt}+{\omega }_{j}+{\varepsilon }_{hjt}$$

Theoretically, the environmental regulations in provinces with higher ventilation coefficient are relatively loose, and the ventilation coefficient is negatively related to environmental regulations. It can be seen from the regression results of the first stage in Table 4 that the coefficient of *vc* is significantly negative, indicating that the results are in line with theoretical expectations.

The estimation results of the second stage show that the coefficient of environmental regulations is significantly positive, indicating that polluting firms are more inclined to locate in areas with more stringent environmental regulation. The main reason is that these areas boast more advanced technological levels and perfect institutional system as well as higher marketization level, so that the benefits of technological innovation can offset the costs of environmental regulations. The stricter the regional environmental regulation, the heavier the punishment given by the government to polluting firms. In the long run, the punishment given by the government can stimulate innovation and increase industry output, which could further enhance the market competitiveness of firms, and in turn make up for the cost of compliance. Moreover, the findings are also supported by facts: as we know, the environmental problems caused by rapid economic growth in the eastern region are more prominent, and thus environmental regulations are relatively strict. Based on the pollution haven hypothesis, the number of newly built polluting firms in the region should be relatively small. However, it can be seen from Table 1 that this number in the eastern region has always been larger than that in the central and western regions. The fact supports the empirical results.

The main explanations for this might be as follows. First, although the eastern region has relatively high environmental regulatory standards, the region has rich human resources, advanced technology, and large market potential. Considering their own long-term development, polluting firms will choose to invest in the region with stricter environment regulation. Second, strict environmental regulations can encourage firms to innovate. Although innovation will increase the cost of the firms, it can improve competitiveness in the long run. Meanwhile, strict environmental regulations can also reduce the labor cost. Third, local governments often give polluting firms equipped with clean production facilities some tax incentives and subsidies in regions with more stringent environmental regulation.

The coefficient of the interaction between environmental regulation and firm pollution intensity is significantly negative, which indicates that the impact of environmental regulations on polluting firms is heterogeneous. For heavily polluting firms, the punishment is heavier and greatly increases the production cost and pollution control cost, and heavily polluting firms are more willing to flow into areas with relatively loose environmental regulations. Firms with more serious pollution are generally listed as key targets of supervision by local governments, and it is difficult for them to evade government supervision, and their pollution control costs will be relatively high. The benefits brought about by technological innovation may not make up for the cost of environmental compliance. Therefore, the heavily polluting firms prefer areas with loose environmental regulation.

The regression results of other control variables show significant differences. The coefficients of economic development level and population size are both significantly positive, which reveals that newly built polluting firms are more likely to enter regions with greater market potential, which is consistent with the “local market effect” theory in the new economic geography. The coefficients of labor scale, labor quality, and labor cost are all significantly negative, which provides additional evidence support that polluting firms are capital-intensive rather than labor-intensive (Broner et al. 2012). At the same time, it is found that provinces with lower labor costs and higher labor quality are more likely to attract polluting firms. The railway infrastructure has a positive impact on firms’ entry decisions; that is to say, polluting firms tend to choose locations with higher accessibility and more convenient transportation. The potential explanation may be that convenient transportation will reduce the transportation costs of firms. The coefficient of land policy is positive but not significant, which implies that firms tend to flow into areas with favorable land policies, but the results show that the government does not support the entry of polluting firms. The technological innovation has a positive relationship with firms entry decisions, which suggests that technological level is a key factor in the location choice of polluting firms. The coefficient of foreign direct investment is significantly negative, which shows that polluting firms are willing to locate in regions with strong foreign market competitiveness.

### Robustness test

#### Basic robustness test

In order to enhance the reliability of the above conclusions, we have conducted the robustness testes from the following point: replacing variables, deleting samples, and changing the set of candidate provinces. First, we replace environmental regulation with industrial sulfur dioxide removal rate and conduct a robustness test. The regression results are shown in column (1) of Table 5. Comparing with the results in Table 4, it can be found that except for the difference in numerical values, the other results are basically the same, indicating that the previous estimation results are robust. Second, because the location choice of state-owned firms is not aimed at maximizing profits, they have to undertake certain social responsibilities, and thus the degree of freedom in location choice is relatively small. Therefore, this paper deletes the sample of state-owned firms. The estimated results in column (2) of Table 5 show that stricter environmental regulations can attract the inflow of newly built polluting firms but will prevent the entry of heavily polluting firms, which is consistent with the previous conclusions. Third, we carry out the robustness test by narrowing the scope of the candidate province set, that is, taking the top 60% and 80% of the province where the polluting firm flows into as the new candidate province set. The estimation results (columns (3) and (4) in Table 5) show that the improvement of environmental regulation standards has increased the probability of entry of newly built polluting firms and reduced that heavily polluting firms. The above analysis shows that the previous regression results are robust.

**Table 5:** Basic robustness test

Variables	(1)	(2)	(3)	(4)
*ER*	0.4932***	1.8293***	1.8366***	1.8160***
	(0.0193)	(0.0981)	(0.1019)	(0.1005)
ER*PI	− 0.1492***	− 0.5732***	− 0.7935***	− 0.7446***
	(0.0275)	(0.2872)	(0.2296)	(0.2247)
Regional fixed effect	Yes	Yes	Yes	Yes
Control variables	Yes	Yes	Yes	Yes
*N*	443570	448320	278800	372900
Log likelihood	− 46891	− 47	− 38620	− 43983
Prob > chi2	0.0000	0.0000	0.0000	0.0000

***, **, and * indicate significant under 1%, 5%, and 10% levels, respectively; figures in the parentheses are standard error.

Own elaboration.

#### Robustness test based on different estimation methods

In order to test the existence of bias by the conditional logit model, we use the Poisson regression model and the negative binomial regression model to conduct the robustness test. The results in Table 6 show that though the absolute value of estimation coefficients is the difference among the three estimation methods, the coefficients are both positive for *ER* and negative for ER*PI, indicating that the conditional logit model is appropriate.

**Table 6:** Robustness test based on different estimation methods

Variables	Poisson regression	Negative binomial regression
*ER*	1.1049***	1.0627***
	(0.0652)	(0.0532)
ER*PI	− 0.2362*	− 0.2134*
	(0.1197)	(0.1274)
Regional fixed effect	Yes	Yes
Control variables	Yes	Yes
*N*	454890	453908
Log likelihood	− 62231	− 63692
Prob > chi2	0.0000	0.0000

***, ** and * indicate significant under 1%, 5%, and 10% levels, respectively; figures in the parentheses are standard error.

Own elaboration.

### Heterogeneous analysis

In the above analysis, we consider firms as homogeneous, and the regression results measure the average effect of environmental regulations on the location choice of polluting firms. However, polluting firms with different characteristics may show different preferences for the strictness of environmental regulations. In this part, samples will be divided according to firm ownership, location, and pollutant to examine the heterogeneous impact of environmental regulations on the location choice of polluting firms.

#### Heterogeneous analysis across ownerships

In China, firms with different ownerships not only have different regulatory and incentive mechanisms but also face completely different treatments in terms of financing preferences and policy support. At the same time, firms with different sources of capital also differ greatly in production technology and innovation capabilities, and this may lead to differences in the sensitivity of the location choice of polluting firms with different ownerships to the intensity of environmental regulations. Table 7 reports the heterogenous analysis results based on different firm ownerships.

**Table 7:** Heterogenous analysis across ownerships

Variables	State-owned firm	Private firm	Foreign-funded firm
*ER*	0.7275	1.7358***	2.2616***
	(0.7050)	(0.1016)	(0.4426)
ER*PI	− 3.1275**	− 0.6723***	1.0892
	(1.2507)	(0.2276)	(1.0512)
Regional fixed effect	Yes	Yes	Yes
Control variables	Yes	Yes	Yes
*N*	12236	408173	31658
Log likelihood	− 1367	− 41974	− 3187
Prob > chi2	0.0000	0.0000	0.0000

***, ** and * indicate significant under 1%, 5%, and 10% levels, respectively; figures in the parentheses are standard error.

Own elaboration.

The regression results in Table 7 show that the impact of environmental regulations on the location choice of polluting firms with different ownerships is quite different. As for state-owned firms, on the one hand, their purpose of operation is not only to maximize profits, but to take part of the responsibility of stabilizing employment and product supply, and the freedom of location choice will be less than that of private firms and foreign-funded firms; on the other hand, compared with firms of other ownerships, they can obtain higher preferential treatment from the government and face lower financing constraints, so the impact of environmental regulations on their location choice is not significant. However, pollution haven effect applies to heavily polluting state-owned firms; that is, polluting firms prefer to choose locations in areas with relatively loose environmental regulations. We find that private firms are willing to enter areas with stricter environmental regulations. The potential explanations for this might be as follows. On the one hand, they have more flexible mechanisms and strong innovation capabilities, and the benefits of technological innovation can make up for the cost of compliance in the long term; on the other hand, the new economic geography theory believes that in the process of location choice, the self-selection effect and the agglomeration effect are equally important. In general equilibrium, firms with similar productivity will cluster together. The eastern coastal areas have the highest degree of marketization and most active private capital; a large number of private firms have gathered here. Therefore, private polluting firms usually choose to flow into the eastern region where environmental regulations are relatively strict. For foreign-funded firms, they boast relatively high level of technological innovation, and thus they prefer factors such as the regional business environment, marketization, and development level. Therefore, environmental regulations have a positive effect on the location choice of foreign-funded polluting firms.

#### Heterogeneous analysis across regions

It is known that the number of newly built polluting firms show an uneven spatial distribution. Therefore, this paper divides the sample into three groups according to administrative divisions, the eastern region, the central region, and the western region, and examines whether there are differences in the impact of environmental regulations on the location of polluting firms in each region (see Table 8).

**Table 8:** Heterogeneous analysis across regions

Variables	Eastern region	Central region	Western region
*ER*	4.1419***	− 3.0125***	0.7323**
	(0.1295)	(0.2469)	(0.3018)
ER*PI	− 1.0324***	0.8752*	0.5874
	(0.2956)	(0.4619)	(0.5425)
Regional fixed effect	Yes	Yes	Yes
Control variables	Yes	Yes	Yes
*N*	281349	103829	66889
Log likelihood	− 22737	− 9764	− 6560
Prob > chi2	0.0000	0.0000	0.0000

***, **, and * indicate significant under 1%, 5%, and 10% levels, respectively; figures in the parentheses are standard error.

Own elaboration.

The regression results in Table 8 show that environmental regulations increase the possibility of polluting firms in the eastern and western regions to choose the corresponding provinces but reduces that in the western region. The possible reason for this difference is that the eastern region boast favorable business environment, strong innovation capabilities, and abundant human capital stock. Even if the environmental regulations are getting stricter in this region; the profits brought about by innovation can make up for the compliance costs brought about by environmental regulations. Firms choose to locate in the central region mainly because the environmental compliance cost in this region is relatively lower than that in the eastern region. Since the innovation capabilities of these firms are insufficient, once the environmental regulation is getting stricter, some firms will choose to flow into these regions where the environmental regulation is relatively loose. The western region is rich in energy and mineral resources, and most firms choose here are generally resource-dependent ones. For the purpose of protecting the natural resources, local government will raise the standards of environmental regulations, but resource-dependent polluting firms still prefer to flow into the region.

#### Heterogeneous analysis across pollutants

Additionally, this paper divides polluting firms into air polluting ones and water-polluting ones in order to distinct location patterns of firms with different pollutants. Due to different forms and traceability of different pollutants, there are also differences in the impact of environmental regulations on the location choice of firms discharging different pollutants (see Table 9).

**Table 9:** Heterogeneous analysis across pollutants

Variables	Air polluting firms	Water-polluting firms
*ER*	2.3824***	1.1416***
	(0.1409)	(0.1388)
ER*PI	− 0.8341***	− 1.2513***
	(0.2697)	(0.4098)
Regional fixed effect	Yes	Yes
Control variables	Yes	Yes
*N*	238106	213961
Log likelihood	− 24359	− 22140
Prob > chi2	0.0000	0.0000

***, **, and * indicate significant under 1%, 5%, and 10% levels, respectively; figures in the parentheses are standard error.

Own elaboration.

As shown in Table 9, the probability of air polluting firms entering areas with strict environmental regulations is higher than that of water-polluting firms, which is mainly related to the difference in the difficulty of the supervision and regulation of the two pollutants. Compared with air pollutants, water pollutants are easier to monitor and track, and it is difficult for water-polluting firms to evade environmental pollution responsibilities. Faced with the same level of environmental regulations, water-polluting firms will bear more pollution costs. Therefore, water-polluting firms are more inclined to choose locations in areas with relatively loose environmental regulations. For heavily pollution firms with both pollutants, strict environmental regulations will prevent them from entering.

## Mechanism

The above research shows that environmental regulations will affect the location choice of newly built polluting firms, so what is the transmission mechanism of the impact? In the theoretical analysis part, it is discussed that environmental regulations can influence the location choice of polluting firms by affecting the regional technological innovation capability and labor cost. In this part, the impact mechanism will be tested. This paper uses the mediation effect model to examine the transmission path of the impact of environmental regulations on the location choice of newly built polluting firms. The test model can be expressed as:

11 $${var}_{jt}={a}_{0}+{a}_{1}{ER}_{jt}+{a}_{2}{X}_{jt}+{\delta }_{jt}$$

12 $$Prob\left({Y}_{hjt}=1\right)={b}_{1}{var}_{jt}+{b}_{2}{ER}_{jt}+{b}_{3}{ER}_{jt}*{PI}_{ht}+{b}_{4}{X}_{jt}+{\omega }_{j}+{\epsilon }_{hjt}$$

where ${var}_{jt}$ stands for technological innovation capability ${tec}_{jt}$ and labor cost ${wage}_{jt,}$ respectively. Likewise, we adopt green patent indicators and average wage of employees as the proxy variable of technological innovation capability and labor cost, and the definitions of other variables are the same as above. Equations (11) and (12) are independent from each other. First, significance of the coefficient ${a}_{1}$ in Eq. (11) is tested, and second, significance of the coefficient ${b}_{1}$ in Eq. (12) is tested. If both ${a}_{1}$ and ${b}_{1}$ are significant, it indicates that environmental regulation can affect the location choice of polluting firms by changing the technological innovation capabilities and labor cost of regions. We conduct OLS regression in Eq. (11) and conditional logit regression in Eq. (12). The regression results are shown in Table 10.

**Table 10:** Mechanism test

Variables	OLS regression		Conditional logit regression	
	ln*tec*	ln*wage*	ln*tec*	ln*wage*
ln*tec*			0.0642***	
			(0.0257)	
ln*wage*				− 1.5624*** (0.0741)
*ER*	0.1096***	− 0.1578**	2.0180***	2.4724***
	(0.0073)	(0.1471)	(0.0627)	(0.0902)
ER*PI			− 0.6158**	− 0.7328***
			(0.2390)	(0.0628)
_*cons*	− 25.0440***	3.0554***		
	(0.0534)	(0.0983)		
Regional fixed effect	No	No	Yes	Yes
Control variables	Yes	Yes	Yes	Yes
*N*	300	300	451480	451260
Log likelihood			− 47340	− 47320
Prob > chi2	0.0000	0.0000	0.0000	0.0000

***, **, and * indicate significant under 1%, 5%, and 10% levels, respectively; figures in the parentheses are standard error.

Own elaboration.

This paper draws on the green patent classification method of *China Green Patent Statistics Report (2014–2017)*; obtains patent data of 30 provinces, municipalities, and autonomous regions in China between 2009 and 2018 from patent search and service platform of the National Intellectual Property Administration by keywords such as pollution control, pollution treatment, environmental materials, alternative energy, energy saving and emission reduction, recycling, and new energy; and compiles the provincial-level green patent indicators. The average wage of employees obtained from the *China Statistic Yearbook* is appropriate for the two following reasons. First, the average wage of employees refers to the average per capita wage during a certain period of time for employed persons. It shows the general level of wage income during a certain period of time and it’s one major indicator to reflect the wage level. Second, the average wage of employees we have selected is average wage of employed persons in urban private units, and this variable doesn’t include the wage of government workers. Therefore, there is no endogenous problem between environmental regulation and the average wage of employees.

From column (1) of Table 10, it is found that the coefficient of $ER$ is significantly positive, indicating that environmental regulations can promote the improvement of the green technology innovation, which is consistent with the Porter hypothesis; namely appropriate environmental regulations can stimulate technological innovation. When local governments raise environmental regulation standards in order to protect the regional environment, polluting firms considering long-term development may choose to improve their own green technology innovation instead of moving to areas with looser environmental regulations. The endogenous growth theory believes that endogenous technological progress is the decisive factor for sustained economic growth, and it is also an important factor that determines whether firms can survive in an increasingly fierce competitive environment. When a firm improves its innovation capability, it cannot only enhance its market competitiveness but also increase the scale effect. Therefore, when the regional environmental regulations standards are improved, even if the production costs of firms increase in the short term, the benefits of firm innovation can offset the costs of environmental regulation in the long run, and firms will choose to innovate locally rather than emigrate.

It can be seen from column (3) of Table 10 that the coefficient of technological innovation level is significantly positive, which suggests that newly built polluting firms tend to flow into regions with higher technological innovation capabilities. The technology spillover effect in the new economic geography theory shows that if the information on new technologies, new products, and new processes in a region is easier to flow and access, then firms clustered in the region will enjoy positive externalities. The high concentration of firms not only indicates that the region has a large market potential but also that the region will have relatively rich human resources. At the same time, regions with high technological innovation capabilities have relatively high levels of green technology, which can significantly reduce the pollution control costs of polluting firms in the region. Therefore, regions with a higher level of technology are more attractive to polluting firms due to their greater market potential, lower pollution control costs, and abundant talent resources. Through the above analysis, we know that environmental regulation can affect the location choice of polluting firms by changing the technological innovation capabilities of regions.

From column (2) of Table 10, it is found that the coefficient of *ER* is significantly negative, indicating that environmental regulations can reduce labor cost. The potential explanations for this might be as follows. On the one hand, with the increasing intensity of environmental regulation, polluting industries will begin to limit production or even withdraw from the market, leading to a decrease of the average wage. On the other hand, the production cost and pollution control cost of firms facing environmental regulation will increase, and firm’s profits will decrease under the condition that the market demand remains unchanged; thus, firms will reduce the real wage. It can be seen from column (4) of Table 10 that the coefficient of wage level is significantly negative, which suggests that newly built polluting firms tend to flow into regions with lower labor cost. Through the analysis, it is learned that environmental regulation can affect the location choice of polluting firms by influencing the regional wage level.

## Conclusions

With the increasingly prominent environmental problems, state and local governments have issued various environmental policies in order to control the trend of environmental deterioration. But will the implementation of these policies affect the location choice of polluting firms? Will it affect the development of the regional industrial? This paper incorporates environmental factors into the new economic geography model and adopts the conditional logit model to empirically test the impact of environmental regulations on the location of polluting firms by using the data of newly built polluting firms from 2009 to 2018. It is found that (1) the strict regional environmental regulation has increased the probability of polluting firms entering but hindered the inflow of heavily polluting firms. (2) The heterogeneous analysis find that environmental regulations have no significant impact on the location choice of state-owned polluting firms, but private firms and foreign-funded firms tend to flow into areas with stricter environmental regulations; environmental regulation has a positive impact on the location choice of polluting firms in the eastern and western regions but negative on that in the central region. At the same time, we find that air-polluting firms are more likely to enter areas with strict environmental regulations than water-polluting firms. (3) Through further mechanism analysis, it is verified that environmental regulations can improve the green technology innovation and cut labor cost to affect the location and layout of polluting firms. This paper also conducts a series of robustness analyses by using different estimation strategies, and the analyses show that the estimation results are robust.

The findings of this paper are of great practical significance for making a more effective environmental policy. (1) When improving environmental standards, local governments may worry about losing local firms or hindering the inflow of firms from other regions, which may result in the failure to implement environmental policies effectively. The research results in this paper show that higher environmental regulatory standards are attractive to polluting firms. Therefore, appropriate environmental policies can achieve a win–win situation for environmental governance and industrial economic development. (2) The research results of this paper show that the location choice of polluting firms is largely determined by whether the increase in revenue from technological innovation in the region exceeds the cost of environmental regulations. The implementation of environmental regulations could be supplemented by necessary green technological innovation incentive policies.

(3) The heterogeneous analysis shows that there are significant differences in the impact of environmental regulations on the location of firms across regions, ownerships, and pollutants. When making environmental policies, it is necessary to fully consider the economic development level, development stage, and environmental carrying capacity of different regions and formulate differentiated environmental policies for different firms in different regions.

It is worth noting that this research may suffer from some limitations. We only study the impact of the environmental regulations on the location choice of newly build polluting firms based on the province level. In the future, it can also be expanded from the following aspects: we can take the city as the spatial unit to study how the environmental regulations influence the location choice of polluting firms; the future study can consider the mechanism problems.

## Data Availability

The datasets used and/or analyzed during the current study are available from the corresponding author on reasonable request.

## Footnotes

1 The 11 heavily polluting industries include the following sectors: Paper Making and Paper Products; Agricultural and By-Product Processing; Chemical Materials and Products Manufacturing; Textile; Smelting and Pressing of Ferrous Metals Products; Food Manufacturing; Electric Power and Heating Power Production and Supply; Leather, Fur, and Coat Products Manufacturing; Petroleum processing and Coking Plant; Nonmetallic Minerals Products; and Smelting and Pressing of Nonferrous Metals.
